# Natural products, including a new caboxamycin, from *Streptomyces* and other *Actinobacteria* isolated in Spain from storm clouds transported by Northern winds of Arctic origin

**DOI:** 10.3389/fchem.2022.948795

**Published:** 2022-11-03

**Authors:** Aida Sarmiento-Vizcaíno, Jesús Martín, Francisco Javier Ortiz-López, Fernando Reyes, Luis A. García, Gloria Blanco

**Affiliations:** ^1^ Departamento de Biología Funcional Área de Microbiología Universidad de Oviedo, Oviedo, Spain; ^2^ Instituto Universitario de Oncología del Principado de Asturias, Universidad de Oviedo, Oviedo, Spain; ^3^ Instituto de Investigación Sanitaria del Principado de Asturias (ISPA), Universidad de Oviedo, Oviedo, Spain; ^4^ Fundación MEDINA Centro de Excelencia en Investigación de Medicamentos Innovadores en Andalucía, Granada, Spain; ^5^ Departamento de Ingeniería Química y Tecnología del Medio Ambiente Área de Ingeniería Química Universidad de Oviedo, Oviedo, Spain

**Keywords:** caboxamycin B, antibiotic, antitumor, streptomyces, micromonospora, nocardiopsis, actinomycete

## Abstract

Actinobacteria, mostly *Streptomyces* species, are the main source of natural products essential in medicine. While the majority of producer microorganisms of secondary metabolite are reported from terrestrial or marine environments, there are limited reports of their isolation from atmospheric precipitations. Clouds are considered as atmospheric oases for microorganisms and there is a recent paradigm shift whereby atmospheric-derived Actinobacteria emerge as an alternative source for drug discovery. In this context, we studied a total of 18 bioactive Actinobacteria strains, isolated by sampling nine precipitation events with prevailing Northern winds in the Cantabrian Sea coast, Northern Spain. Backward trajectories meteorological analyses indicate that air masses were originated mostly in the Arctic Ocean, and their trajectory to downwind areas involved the Atlantic Ocean and also terrestrial sources from continental Europe, and in some events from Canada, Greenland, Mauritania and Canary Islands. Taxonomic identification of the isolates, by 16S rRNA gene sequencing and phylogenetic analyses, revealed that they are members of three Actinobacteria genera. Fifteen of the isolates are *Streptomyces* species, thus increasing the number of bioactive species of this genus in the atmosphere to a 6.8% of the total currently validated species. In addition, two of the strains belong to the genus *Micromonospora* and one to genus *Nocardiopsis*. These findings reinforce a previous atmospheric dispersal model, extended herein to the genus *Micromonospora*. Production of bioactive secondary metabolites was screened in ethyl acetate extracts of the strains by LC-UV-MS and a total of 94 secondary metabolites were detected after LC/MS dereplication. Comparative analyses with natural products databases allowed the identification of 69 structurally diverse natural products with contrasted biological activities, mostly as antibiotics and antitumor agents, but also anti-inflammatory, antiviral, antiparasitic, immunosuppressant and neuroprotective among others. The molecular formulae of the 25 remaining compounds were determined by HRMS. None of these molecules had been previously reported in natural product databases indicating potentially novel metabolites. As a proof of concept, a new metabolite caboxamycin B (1) was isolated from the culture broth of *Streptomyces* sp. A-177 and its structure was determined by various spectrometric methods. To the best of our knowledge, this is the first novel natural product obtained from an atmospheric *Streptomyces*, thus pointing out precipitations as an innovative source for discovering new pharmaceutical natural products.

## Introduction

Members of the Phylum Actinobacteria, also known as actinomycetes, are major producers of secondary metabolites of medical and biotechnological use, being the *Streptomyces* genus the most prolific source of natural products of pharmaceutical interest. At present, there is an urgent need of new secondary metabolites, essentially antibiotics to combat pathogenic resistant bacteria, but also compounds with applications as anticancer agents, antivirals, antiparasitics and immunosuppresants, among others (Ramirez-Rendon et al., 2022).

New trends in natural products discovery are now focused in the search for novel producers in the less explored environments of our planet, including deep-sea habitats and the atmosphere. Regarding the biogeography of the *Streptomyces* genus, it is known that they are ubiquitous since they are widespread not only in terrestrial habitats, as was established in the last century, but also in oceanic and atmospheric environments (Braña et al., 2015; Sarmiento-Vizcaíno et al., 2017a). An atmospheric dispersal model, coupled with the main Earth hydrological cycle, was proposed to explain the biogeography of *Streptomyces* species (Sarmiento-Vizcaíno et al., 2016).

Highly diverse bioactive *Streptomyces* and *Nocardiopsis* species were isolated from multiple precipitation events happened in the Cantabrian Sea Coast, Northern Spain, with prevalent Western and North Western winds (Sarmiento-Vizcaíno et al., 2021, 2018). It has been suggested that during different precipitation events and by changing the latitude of the sampling place, it is possible to isolate a great diversity of Actinobacteria producing a remarkable reservoir of natural products with relevant biological activities, thus revealing the pharmaceutical and biotechnological potential of the atmosphere as a relevant source for natural products discovery (Sarmiento-Vizcaíno et al., 2021).

As a result of a multidisciplinary approach, we explored here the phylogenetic and biosynthetic diversity of Actinobacteria obtained in the Cantabrian Sea Cost, Spain, over 3 years’ time in precipitation events with Northern winds, the still remaining unexplored source of precipitations in this geographical region. This culture-dependent approach involved taxonomical and phylogenetic analyses as well as meteorological analyses. Antimicrobial assays, metabolic profiling by LC-UV-MS, followed by identification by comparison to natural products databases, purification and structural elucidation, were used to uncover the biosynthetic diversity of these atmospheric-derived Actinobacteria.

## Materials and methods

### Sampling of atmospheric precipitations

**FIGURE 1:**
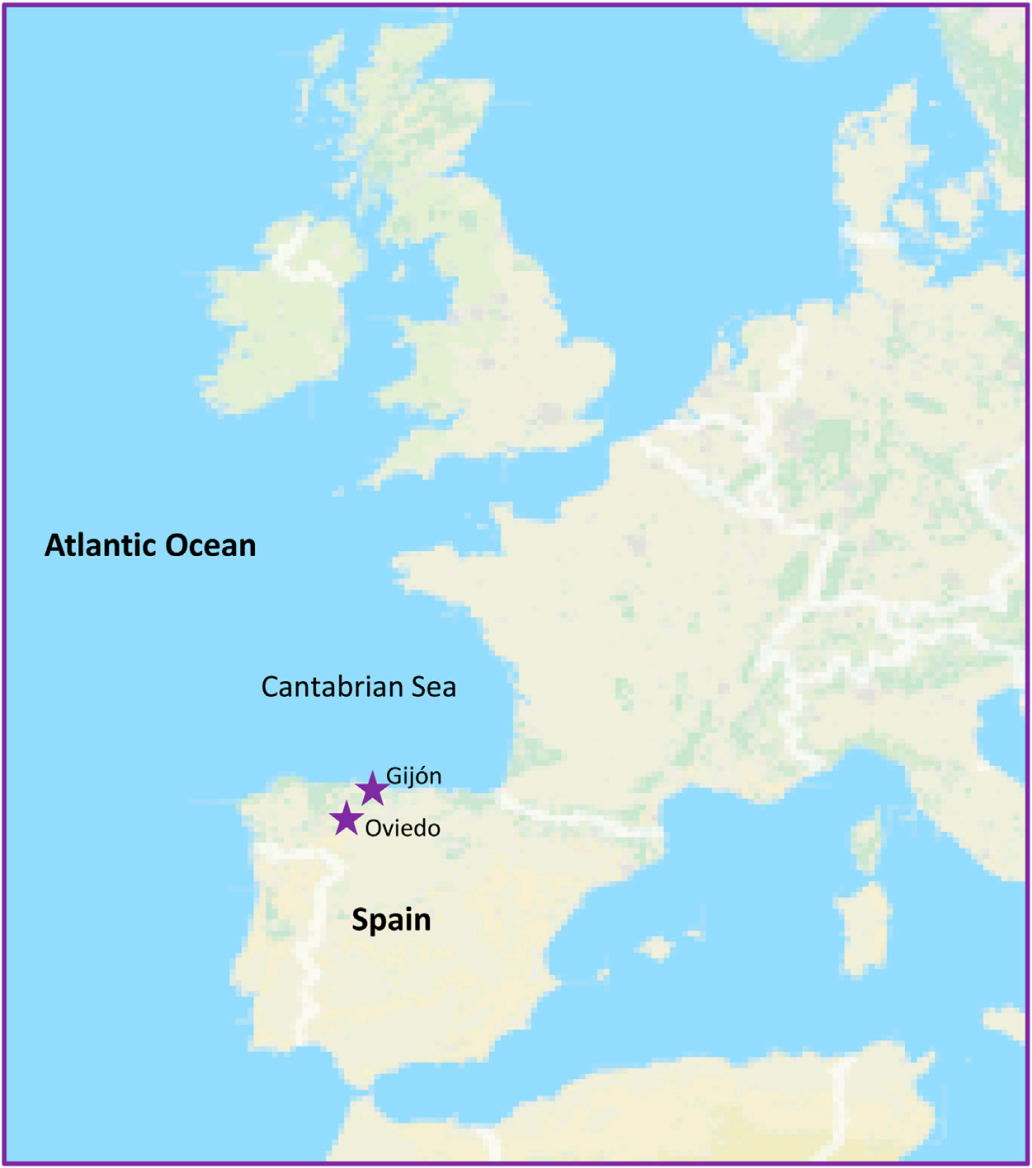
Sampling locations. Overview of the Western Atlantic Ocean and the sampling locations in Northern Spain indicated by stars.

Rainwater, hailstone and snow samples were collected within the years 2013–2016 in Northern Spain, at the Asturias Cantabrian Sea coastal region, a very wet and rainy region whose climate is influenced by the Atlantic Ocean (Figure 1). Samples of 2–3 ml were taken in sterile recipients mainly at the coastal locality of Gijón (43° 32′ N, 5° 39′ W), but also in Oviedo (43° 21′ N, 5° 52′ W), and plated on selective media as has been described (Braña et al., 2015; Sarmiento-Vizcaíno et al., 2016). During most of the precipitation events sampled the prevailing wind direction was Northern.

### Actinobacteria strains isolation and culture

Actinobacteria strains were isolated after plating of atmospheric samples on selective agar media, prepared with cycloheximide (80 μg mL–1) as antifungal and nalidixic acid (20 μg mL–1) as anti-Gram negative bacteria, using MOPS BLEB 1/6 (Oxoid) basal medium as previously reported (Sarmiento-Vizcaíno et al., 2016). The selection plates were prepared either with distilled water or with a supplement of 3.5% NaCl. Colonies were selected based on different morphological features and pigment production on R5A agar plates after incubation for 2–3 weeks at 28 °C. Pure cultures were conserved in 20% glycerol at −20°. MOPS BLEB 1/6 was also used as the basal medium for halotolerance studies, adding NaCl at 0, 3.5, 7.0, and 10.5% (w/v) final concentrations. For secondary metabolite production R5A medium was used as previously described (Sarmiento-Vizcaíno et al., 2018).

### Air mass backward trajectories analyses

Backward trajectories of the air masses were generated using the HYSPLIT model (Hybrid Single Particle Lagrangian Integrated Trajectory) from the Global Data Assimilation System of National Oceanic and Atmospheric Administration, USA (Stein et al., 2015) to estimate the long-range transport journey of air masses that originated the precipitation events herein studied. Five-day backward trajectories were obtained using the NOAA model (http://ready.arl.noaa.gov/hypub-bin/trajtype.pl?runtype=archive) to track the transport pathways of air masses and determine the origin of diverse air parcels. Sampling locations were used as the backward trajectory start point with altitudes over the sea level of 30, 1,000 and 3,000 m (Gijón), and 300, 1,000 and 3,000 m (Oviedo) as previously reported (Sarmiento-Vizcaíno et al., 2021).

### Antimicrobial bioassays

To determine the antimicrobial activities of isolates, agar diffusion methods were used against a panel of the following indicator microorganisms: the Gram-positive bacteria *Micrococcus luteus* ATCC 14452 and *Streptomyces* 85E ATCC 55824, the Gram-negative *Escherichia coli* ESS, and the yeast *Saccharomyces cerevisiae var. Carlsbergensis*. Analyses were performed in TSA1/2 (Merck) against bacteria and in Sabouraud 1/2 (Pronadisa) against yeast, and bioassays were carried out both with agar plugs (7 mm diameter) and also with 6-mm-diameter AA Discs (Whatman), loaded with ethyl acetate extracts of bioactive isolates, as previously reported (Sarmiento-Vizcaíno et al., 2021). Agar plugs assays detect all diffusible compounds produced by actinobacterial strains, both polar and apolar, whereas the AA discs bioassays only detect diffusible apolar molecules extracted with ethyl acetate.

### 16S RNA analysis identification and phylogenetic analysis

DNA was extracted with a microbial isolation kit (Ultra Clean, MoBio Laboratories, Inc.) for taxonomic identification of the strains, using standard methods for checking the purity (Russell and Sambrook, 2001). Partial 16S rRNA gene sequences of the bacterial strains were obtained by PCR amplification using the 616V (forward) and 699R (reverse) primers (Arahal et al., 2008) (Braña et al., 2015), using the BLAST program (Basic Local Alignment Search Tool) against the NCBI (National Centre for Biotechnology Information). The nucleotide sequences were compared to sequences in databases, submitted and deposited in the EMBL database with accession numbers OL587568-OL587585. Phylogenetic analysis of the strains based on 16S rRNA gene sequences was performed as previously reported (Sarmiento-Vizcaíno et al., 2021).

### Chromatographic analysis

Plugs of R5A plates (about 7 ml) were extracted using ethyl acetate in neutral and acidic (with 1% formic acid) conditions. After evaporation, the organic fraction residue was redissolved in 100 µL of a mixture of DMSO and methanol (50:50). The analyses of the samples were performed by reversed phase liquid chromatography as previously described (Braña et al., 2015; Sarmiento-Vizcaíno et al., 2016).

### Identification of compounds by LC-UV-Vis and LC-UV-HRMS analyses

Samples were first analyzed and evaluated using an in-house HPLC-UV-Vis database. LC-UV-HRMS analyses were carried out as has been described (Pérez-Victoria et al., 2016; Sarmiento Vizcaíno et al., 2018) and major peaks in each chromatogram were searched against the MEDINA’s internal database and also against the Dictionary of Natural Products (DNP) (Chapman and Hall, 2015).

### Purification of novel compounds

For purification of the secondary metabolites produced, *Streptomyces* sp. A-177 was cultured in 40 Erlenmeyer flasks (250 ml), each containing 50 ml of R5A medium supplemented with 3%. DMSO inoculated with spores and incubated in an orbital shaker at 28°C and 250 rpm during 7 days. The cultures were centrifuged and extracted with ethyl acetate acidified with 1% formic acid. The extracts were dried and the residue was subsequently redissolved in a small volume of acetonitrile and DMSO (1:1). The same peaks were also found in the organic extract of the culture pellets, which were dried and redissolved in the same way. The desired compounds were purified by preparative HPLC using a SunFire C18 column (10 μm, 10 × 250 mm, Waters). The purification was performed in two steps. The mobile phase was a mixture of 90% acetonitrile and TFA 0.1% in the first step and 80% acetonitrile and TFA 0.1% in the second one, in isocratic conditions at 5 ml/min. In both cases, the solutions containing the collected peaks were evaporated in rotavapor and finally lyophilized, resulting in 3.9 mg of caboxamycin B (**1)**.

### LC-HRMS and NMR analyses

HRMS spectra (ESI-TOF) were acquired using a Bruker maXis QTOF mass spectrometer coupled to an Agilent 1,200 Rapid Resolution HPLC. NMR spectra were recorded at 297 K on a Bruker Avance III spectrometer (500 and 125 MHz for 1H and 13C, respectively) equipped with a 1.7 mm TCI MicroCryoProbeTM. 1H and 13C chemical shifts were reported in ppm using the signals of the residual solvent as internal reference (δH 2.50 and δC 39.5 ppm for DMSO-d6).

## Results

### Selection of bioactive Actinobacteria from precipitation events in Northern Spain

The strains studied herein were isolated and characterized in Spain by sampling multiple precipitation events, as previously reported (Sarmiento-Vizcaíno et al., 2021). After a dereplication process, involving antimicrobial activity, phenotypical features, metabolic profiling and meteorological analyses, 18 morphologically different bioactive strains, isolated in precipitations events with prevalent Northern winds, were here selected. To analyze antibiotic production, the isolates were initially screened for antimicrobial activity against bacteria and fungi as indicator microorganisms (Table 1), using agar diffusion methods as recently reported (Sarmiento-Vizcaíno et al., 2021). Table 1A shows the results of bioassays with agar plugs and Table 1B displays the results of ethyl acetate extracts using agar diffusion with AA discs. Most of the strains display strong antibiotic activities against Gram-positive bacteria, such as *M. luteus* and *Streptomyces* 85E, whereas 10 strains were active against the Gram-negative *E. coli* ESS and seven strains against the yeast *S. cerevisiae*.

**TABLE 1:** Antibiotic activities of Actinobacteria against a panel of Gram-negative, Gram-positive bacteria and fungi.

	A. Agar plugs				B. Ethyl acetate extracts AA discs (Neutral/Acid)		
Strain	*E. coli*	*M. luteus*	*Streptomyces* 85E	*S. cerevisiae*	*E. coli*	*M. luteus*	*S. cerevisiae*
A-4	-	16	34	13	ND	ND	ND
A-28	-	15	15	20	-	16/17	-/9
A-104	14	16	18	12	17/29	20/22	11/9
A-105	-	22	25	-	9/-	25/25	-
A-106	-	12	25	-	-	-/8	-
A-120	-	23	-	22	-	16/10	11/10
A-121	-	12	11	-	-	19/16	-
A-123	-	21	19	16	13/-	28/20	-
A-124	20	27	27	-	23/16	12/13	12/12
A-125	-	19	12	-	12/24	20/20	-
A-145	-	21	27	-	-	24/24	-
A-159	-	30	15	-	-	30/26	-
A-163	-	14	15	-	-/9	13/16	-
A-176	-	-	15	-	-/16	16/14	-
A-177	-	10	8	-	-/11	17/17	-
A-182	-	-	17	-	-/12	-/10	-
A-183	-	18	25	-	21/11	22/18	15/18
A-199	-	-	-	-	-	10/9	-

Antimicrobial activities were estimated as the zones of complete inhibition (diameter in mm). Table 1A. Assays performed with agar plugs from solid cultures. Table 1B. Antibiotic activities of ethyl acetate extracts of the cultures. 7 ml of culture, in neutral and acidic conditions, resuspended in 50μL of DMSO-methanol (1:1) from which 15 μl were loaded onto AA discs. Before applying to the indicator strain culture, the discs were allowed to fully dry.

### Air mass backward trajectories analyses

Selected bioactive strains were isolated in Northern Spain during years 2013, 2015 and 2016 in nine precipitation events in which the prevailing wind direction was Northern. Samples were mostly collected in Gijón, sampling place, during eight events of rainfall and hailstone, and one snow event was sampled in Oviedo.

**FIGURE 2:**
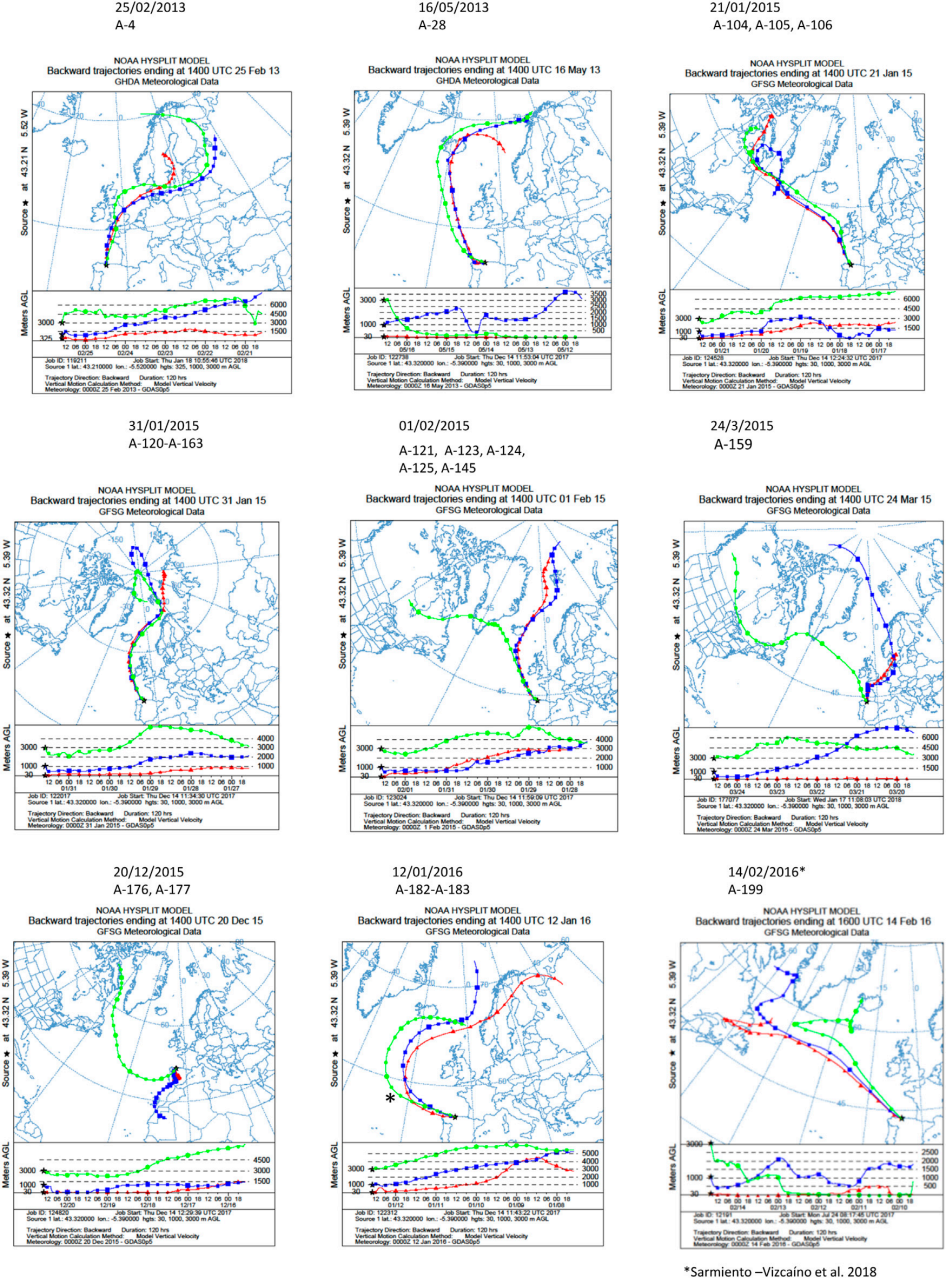
Five-day backward trajectories of air masses generating the storms that caused the precipitation events. They were calculated with the NOAA HYSPLIT Model with three different transects with different arriving height as previously reported (Sarmiento-Vizcaíno et al., 2018). The sampling locations were used as the backward trajectory start point with altitudes over sea level of 30, 1,000 and 3,000 m (Gijón), 300, 1,000 and 3,000 m (Oviedo). Black asterisks indicate the sampling places. The strains isolated and the dates of the precipitation events are shown on the top of the figures.

NOAA meteorological analyses were addressed to estimate the origin and trajectories of the air masses that caused different precipitation events in which the isolate were obtained. As shown in Figure 2, 5 days HYSPLIT backward trajectories were performed in the sampling locations at three different arriving heights. In all events the air masses were transported by prevalent Northern Winds, and the backward trajectories showed that air masses were mainly originated in the Arctic Ocean, close to the North Pole, and traveled toward South Europe, such as the sampling place in North Spain, thus revealing a main oceanic route from the Arctic and the North Atlantic Oceans. In some events, the air masses trajectory also involves terrestrial routes in Europe, Canada and Greenland to downwind areas.

### Taxonomic identification, phylogenetic analyses and distribution of bioactive isolates

The nucleotide sequences of the 16S rRNA gene fragments of bioactive strains were deposited in the EMBL database and accession numbers are displayed in Table 2, which also shows the closest phylogenetic relatives with indication of their isolation source. A generalized feature in all strains is their salt tolerance (Table 2). Most of the isolates tolerate NaCl concentrations, up to 7%, which is in agreement with previous reports (Sarmiento-Vizcaíno et al., 2021, 2018).

**TABLE 2:** Phylogenetic diversity of Actinobacteria isolates.

							
Strain	EMBL A. N		NaCl %		Closest homologue	A. N	% Homology (bp)	Isolation source	References
*Streptomyces* sp. A-4	OL587568		3.5		*Streptomyces xanthophaeus* NBRC 12829T	AB184177	100 (1,012/1,012)	Soil and rizosphere, India	Singh et al. (2016)
*Streptomyces* sp. A-28	OL587569		<3.5		*Streptomyces hygroscopicus* NRRL 2387T	AJ391820	100 (982/982)	Soil, Australia; precipitations, Spain	Jensen. (1931); Sarmiento-Vizcaíno et al. (2018)
*Streptomyces* sp. A-104	OL587570		7		*Streptomyces alfalfae* XY25T	NR_147,713	100 (599/599)	Alfalfa rhizosphere, China	She et al. (2016)
*Streptomyces* sp A-105	OL587571		7		*Streptomyces mediolani* NBRC 15427T	AB184674	99,8 (971/973)	*Taxus baccata* roots, Slovakia	Jimenez et al. (2012)
*Streptomyces* sp A-106	OL587572		7		*Streptomyces mediolani* NBRC 15427T	AB184674	99,6 (971/975)	*Taxus baccata* roots, Slovakia	Jimenez et al. (2012)
*Streptomyces* sp. A-120	OL587573		3.5		*Streptomyces geldanamycininus* NRRL 3602T	DQ334781	99,9 (970/971)	Soil, atmospheric precipitations	Goodfelow et al. (2007); Sarmiento-Vizcaíno et al. (2018)
*Nocardiopsis* sp*.* A-121	OL587574		7		*Nocardiopsis alba* DSM 43377T	MN688677	99,9 (993/994)	Honeybees gut, bioaerols, precipitations	Qiao et al. (2012); Pasciak et al. (2014); Sarmiento-Vizcaíno et al. (2021)
*Streptomyces* sp. A-123	OL587575		7		*Streptomyces cyaneofuscatus* NBRC 13190T	AB184860	100	Marine, atmospheric and terrestrial, Spain	Braña et al. (2015); Sarmiento-Vizcaíno et al. (2017a), Sarmiento-Vizcaíno et al. (2018)
*Streptomyces* sp. A-124	OL587576		7		*Streptomyces albiaxialis* NBRC 101002T	AB249952	100 (983/983)	Oil field, Russia	Whitman et al. (2012)
*Streptomyces* sp*.* A-125	OL587577		3.5		*Streptomyces cyaneofuscatus* NBRC 13190T	AB184860	98,8 (964/976)	Marine, atmospheric, terrestrial, Spain	Braña et al. (2015); Sarmiento-Vizcaíno et al. (2017a), Sarmiento-Vizcaíno et al. (2018)
*Streptomyces* sp. A-145	OL587578		7		*Streptomyces mediolani* NBRC 15427T	AB184674	99,9 (964/965)	*Taxus baccata* roots, Slovakia	Jimenez et al. (2012)
*Streptomyces* sp. A-159	OL587579		7		*Streptomyces cyaneofuscatus* NBRC 13190T	AB184860	99,8 (980/982)	Marine, atmospheric and terrestrial, Spain	Braña et al. (2015); Sarmiento-Vizcaíno et al. (2017b), Sarmiento-Vizcaíno et al. (2018)
*Streptomyces* sp A-163	OL587580		<3.5		*Streptomyces sparsogenes* NBRC 13086T	AB184301	98,6 (953/966)	Marine sponge, Japan	Khan et al. (2011)
*Micromonospora* sp. A-176	OL587581		<3.5		*Micromonospora aurantiaca* DSM 43813T	X92,604	99,4 (791/796)	Soil, Germany; deep sea echinoderm, Spain	Tindall et al. (2014)
									Sarmiento-Vizcaíno et al. (2017b)
*Streptomyces* sp. A-177	OL587582		7		*Streptomyces pactum* NBRC 13433T	AB915617	100 (965/965)	Marine sediment, Thailand	Ruttanasutja and Pathom-aree, (2015)
*Streptomyces* sp A-182	OL587583		7		*Streptomyces thermospinosisporus* AT10T	KU141346	100 (973/973)	Dung beetle, South Korea	Kim and Goodfellow. (2002)
*Streptomyces* sp. A-183	OL587584		7		*Streptomyces mediolani* NBRC 15427T	AB184674	99,9 (962/963)	*Taxus baccata* roots, Slovakia	Jimenez et al. (2012)
*Micromonospora* sp. A-199		OL587585		3.5	*Micromonospora arenae* MuizA5ST	EU196563	100 (977/977)	Marine sediment, South Africa	Kirby and Meyers, (2007)

**FIGURE 3:**
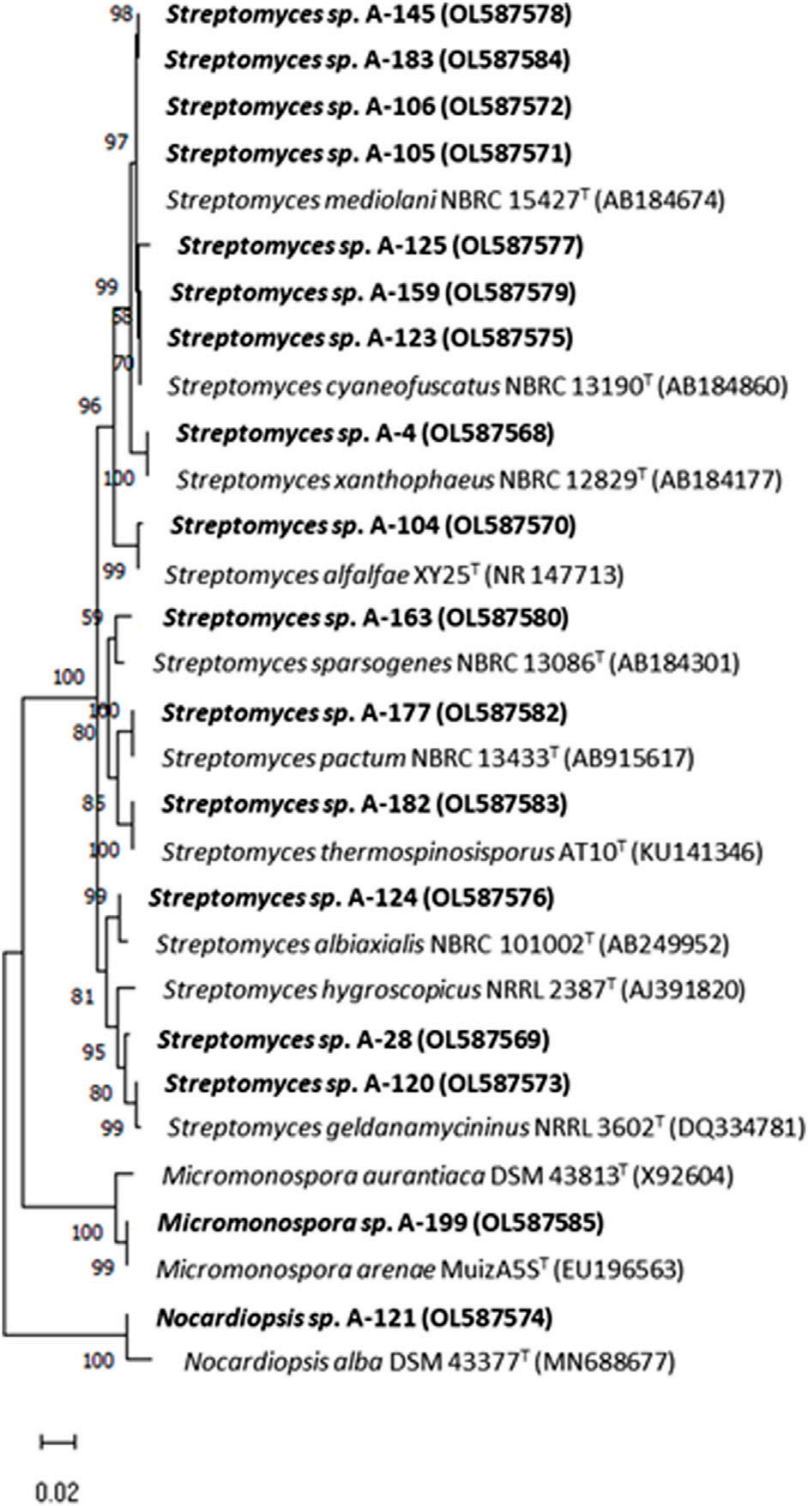
Neighbor-joining phylogenetic tree generated by distance matrix analysis of 16S rRNA gene sequences from atmospheric Actinobacteria, *Streptomyces*, *Micromonospora* and *Nocardiopsis* (highlighted) and their nearest phylogenetic relatives. The numbers on branch nodes indicate bootstrap values (1,000 resamplings; only values > 50% are shown). Bar represents 1% sequence divergence.

The results of the phylogenetic analyses based on 16S rRNA gene alignments, revealed that all 18 strains share 99.4–100% identity with known actinobacterial species, belonging to three different genera among the *Phylum Actinobacteria*. Among 18 studied strains, 15 belonged to the *Streptomyces* genus, having their closest homologues in species previously isolated from terrestrial, marine and atmospheric environments (Table 2), in association with lichens, plants, terrestrial invertebrates (honeybees, beetles), marine macroalgae and marine invertebrates. In addition, two strains belonging to the *Micromonospora* genus were obtained in two precipitation events (Table 2). This genus has been proposed as a source for bioactive natural products discovery (Hifnawy M. S. et al., 2020; Hifnawy M. et al., 2020). Also a strain belonging to the genus *Nocardiopsis* was identified in one precipitation event. A neighbour-joining phylogenetic tree (Figure 3) was built to estimate the relationship between the strains and their nearest phylogenetic relatives.

### Identification of bioactive secondary metabolites

**FIGURE 4:**
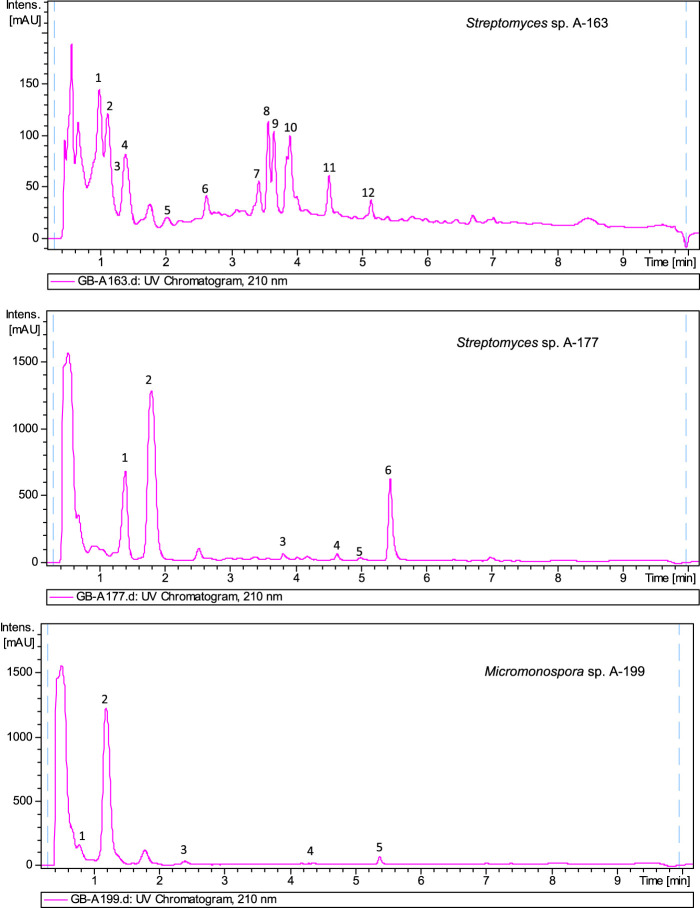
UV_210nm_chromatogram of ethyl acetate extracts from *Streptomyces* sp A-163, *Streptomyces* sp A-177 and *Micromonospora* sp. A-199. Dereplicated components in sample A-163: 1) Hydroxystreptazolin, 2) Streptazolin, 3) Cyclo (leucylprolyl), 4) C_12_H_24_O_4_ (molecular formula not found in the Dictionary of Natural Products for prokaryotes), 5) C_18_H_16_O_4_ (no UV coincidence in the Dictionary of Natural Products for prokaryotes), 6) C_11_H_13_NO_2_ (no UV coincidence in the Dictionary of Natural Products for prokaryotes), 7) C_12_H_20_O_3_ (no UV coincidence in the Dictionary of Natural Products for prokaryotes) (8 and 9) BE 14348B (10) BE 14348D/E (11) WS 9761B (12) Antibiotic W 007. Dereplicated components in sample A-177: 1) Cyclo (phenylalanylprolyl), 2) C_13_H_14_O_4_ (no UV coincidence in the Dictionary of Natural Products for prokaryotes), 3) Alteramide A, 4) Clifednamide A, 5) C_15_H_10_ClNO_4_ new caboxamycin derivative, 6) Caboxamycin B (1). Dereplicated components in sample A-199: (1) Cyclo (4-hydroxyprolylphenylalanyl), (2) N-[2-(1H-Indol-3-yl)-2-oxoethyl]acetamide, (3) C_14_H_12_N_2_O_5_ (molecular formula not found in the Dictionary of Natural Products for prokaryotes), (4) Macrolactin N, (5) Diazepinomicin.

The biosynthetic potential of the strains studied herein was uncovered by metabolite profiling analyses of ethyl acetate extracts of strains. Extracts were analyzed by LC-UV and LC/HRMS in combination with searches in UV and MS databases or the Dictionary of Natural Products (DNP), after generation of a molecular formula of each peek based on HRMS results. Complex metabolic profiles were obtained, with most of the strains producing multiple natural products in R5A medium (Supplementary Material S1). As an example, Figure 4 displays UV_210 nm_ chromatograms corresponding to three samples.

Among a total of 94 secondary metabolites detected after LC/MS dereplication and comparative analyses of ethyl acetate extracts of all strains, 69 were identified as having matches in DNP, as shown in Table 3. The remaining 25 compounds had molecular formulae determined by HRMS not previously reported for any molecule included in natural product databases (see Table 4) and are of great pharmaceutical interest since they might be new natural products and thus candidates for novel drugs discovery. Regarding the previously reported biological activities of known natural products identified, 36 display biological activities as antibiotics, both antibacterial and antifungal, 22 as antitumor/cytotoxic, four anti-inflammatory, three immunosuppresant, two antiviral, two antiparasitic, two neuroprotective, one hepatoprotective one immunostimulator, and other compounds of pharmacological interest (Table 3). They belong to remarkably diverse structural families, such as anthracyclines, angucyclinones, ansamycins, macrolactams, polienes, polyketides, alkaloids, macrolides, phenazines and others.

**TABLE 3:** Identified compounds produced by bioactive *Streptomyces*, *Micromonospora* and *Nocardiopsis* strains and their biological activities.

		
Compound LC/MS	Strain	Biological activities	
1,2-Di (1H-indol-3-yl)ethane/Vibrindole A	A-124	Cytotoxic Al-Zereini et al. (2010), Bell et al., (1994)	
3-Benzylidene-6-(4-methoxybenzylidene)-2,5-piperazinedione	A-121	Unknown	
3-Hydroxyundecanoic acid	A-121	Unknown	
4,5-dihydrogeldanamycin^c^	A- 120	Anticancer Wu et al. (2012)	
4-N-Hydroxy, 1-N-Me 3-Benzylidene-6-isobutylidene-2,5-piperazinedione/ 4″-Methoxy 3-Benzylidene-6-isobutylidene-2,5-piperazinedione/3′-Hydroxy, 1-N-Me 3-Benzylidene-6-isobutylidene-2,5-piperazinedione^a^	A-121	Unknown	
9-(4-Aminophenyl)-3,7-dihydroxy-2,4,6-trimethyl-9-oxononanoic acid	A-104	Unknown	
9-C-Mycarosyl-4-O-demethylpremithramycinone/9-C-Olivosylpremithramycinone/Premithramycin A1/Aranciamycin/Arimetamycin B/8-Demethoxysteffimycin^a^	**A-176**	Antitumor Luzhetskyy et al. (2008)	
10′-Demethoxystreptonigrin	A-124	Unknown	
Alteramide A^c^	A-177	Cytotoxic Shigemori et al. (1992); antifungal Moree et al. (2014)	
Antibiotic TAN 420C	A-28	Antibiotic (Seiichi et al., 1983)	
Antibiotic TAN 420E	A-28	Antibiotic (Seiichi et al., 1983)	
Antibiotic X 14881D	A-4	Antibiotic Maehr et al. (1982)	
Antibiotic W 007	A-163	Antibiotic, antitumor Zhang et al. (2012)	
Bacillcoumacin A/Methyl 3-((4-formylphenyl)amino)-4,7-dihydroxyoct-5-enoate/Methyl phenatate A^a^	A-125	Antibiotic Liu et al. (2016)	
BE 14348D/E	A-163	Estrogen-receptor binding inhibitors Kondo et al. (1990)	
Collismycin A/B	A-183	Antiinflamatory, antibiotic Shindo et al. (1994)	
Collismycin D	A-183	Neuroprotective Garcia et al. (2013)	
Collismycin F	A-183	Unknown	
Clifednamide A	A- 177	Cytotoxic Jiao et al. (2020)	
Cyclo (4-hydroxyprolylphenylalanyl)	**A-199**	Unknown	
Cyclo (leucylprolyl)^b^	Several strains A	Antibiotic, cytotoxic Santos et al. (2015)	
Cyclo (phenylalanylprolyl)	Several strains B	Antitumor, antibiotic Milne et al. (1998)	
Cyclo (phenylalanylvalyl)	**A-176**	Unknown	
Cyclo (prolyltyrosyl)	**A-176**	Antifungal, antiparasitic, anticancer Santos et al. (2019)	
Cyclo (prolyltryptophyl)^c^	A-4, A-145, **A-176**, A-183	Antibacterial Blunt and Munro (2008)	
Cyclo (prolylvalyl)^b^ ^,^ ^c^	A-4, A-183	Antifungal Kumar et al. (2014)	
Cyclo (valylprolyl)^b^	A-145	Antibacterial Alshaibani et al. (2017)	
Cytoxazone	**A-121**	Cytokine modulator Kakeya et al. (1998)	
Dermostatin A	A-104	Antifungal Gordee et al. (1971)	
Dermostatin B	A-104	Unknown	
Diazepinomicin	**A-199**	Antimicrobial Charan et al. (2004)	
Diacetyl-phenazinediol/Dimethyl phenazine-1,6-dicarboxylate	A-183	Unknown	
Divergolide M	A-106	Antibiotic, cytotoxic Ding et al. (2015)	
Espicufolin A	A-145	Antitumor Tietze et al. (2007)	
Geldanamycin^b^	A-120	Antifungal, anticancer, neuroprotective Tadtong et al. (2007)	
Germicidin A^b^	A-182	Spore germination (hypha elongation Aoki et al. (2011)	
Germicidin B^b^/C	A-182	Spore germination (hypha elongation Aoki et al. (2011)	
Glycinocin A	A-28	Antibiotic Corcilius et al. (2018)	
Herbimycin A (Antibiotic TAN 420F)	A-28	Antibiotic, antitumor Garcia et al. (1991)	
Hydroxystreptazolin	A-163	Cytotoxic Puder et al. (2001)	
Ilamycin (rufomycin) A/C1/C2^b^ ^,^ ^c^	A-159	Cytotoxic Ma et al. (2017)	
Ilamycin B1^c^	A-159	Unknown	
Inthomycin A/B/C	A-125	Antibiotic Kim et al. (2020)	
Macbecin I	A-28	Antibiotic, antitumor Martin et al. (2008)	
Macrolactin N	**A-199**	Antibiotic Yoo et al. (2006)	
Methylsulfomycin I^b^	A-105	Antibiotic Vijaya Kumar et al. (1999)	
MH 031	A-182	Hepatoprotective Itoh et al. (1991)	
MDN-0097	A-183	Unknown	
N-[2-(1H-Indol-3-yl)-2-oxoethyl]acetamide	A-4, **A-199, A-176**	Antibacterial, antifungal, antitumor Smaoui et al. (2012)	
N-(2-Hydroxybenzoyl), 1-Acetyl-serinol	A-182	Antiviral Matzzotta et al. (2021)	
Nb-Acetyltryptamine	**A-176**	Anti-neuroinflammatory Kim et al. (2016)	
Neopluramycin	A-145	Antibiotic, antitumor Kondo et al. (1970)	
NFX 2/2-Methyl-2,5,6-bornanetriol	**A-121**	Unknown	
Nigericin^b^	A-28	Antibiotic, cytotoxic Wang et al. (2017)	
Nybomycin	A-124	Antibiotic, cytotoxic Zakalyukina et al. (2019)	
Pentalenolactone/Pentalenolactone A/Pentalenolactone B/Pentalenolactone P	A-104	Antibiotic Tetzlaff et al. (2006)	
Pentalenolactone H/Pentalenolactone O	A-104	Antibiotic Zhu et al. (2011)	
Pentalenolactone I	A-104	Inmunosuppressant Uyeda et al. (2001)	
Phaeochromycin E	A-106	Antiinflammatory Graziani et al. (2005)	
Phencomycin	A-183	Antiinflammatory Graziani et al. (2005), Antibacterial Hifnawy et al. (2020a)	
Prodigiosin 25b^c^	A-182	Antibiotic Harashima et al. (1967)	
Pulvomycin	A-123	Antibiotic Parmeggiani et al. (2006)	
Salbomycin	A-28	Antibiotic, antihelmintic, anticancer, immunosuppressive, antiinflammatory, antiviral Gui et al. (2019)	
Saptomycin A/β-Indomycinone	A-145	Antibiotic, antitumor Abe et al. (1993)/Cytotoxic Tsukahara et al. (2014)	
Sceliphrolactam	A-125	Antifungal Kim et al. (2021)	
Streptazolin	A-163	Immunostimulator Perry et al. (2015)	
Undecylprodigiosin^b^	A-182	Antibiotic, cytotoxic Petrovic et al. (2017), Immunosuppressant Songia et al. (1997)	
Venturicidin A	A-125	Antibiotic adjuvant Yarlagadda et al. (2020)	
Venturicidin C	A-125	Antifungal Shaaban et al. (2014)	
WS 9761B	A-163	Androgen-receptor antagonist Hori et al. (1993)	

^a^ The asterisk means that more than one compound was identified.The highlightened strains correspond to Micromonospora sp. and the underlined to Nocardiopsis sp., the rest are all *Streptomyces* species.

A: A-4, A-104, A-106, **A-121**, A-123, A-124, A-125, A-145, A-163, A-176, A-182, A-183.

B: A-177, A-121, A-123, A-134, A-145, A-183.

^b^ Sarmiento-Vizcaíno et al., 2018.

^c^ Sarmiento-Vizcaíno et al., 2018.

**TABLE 4:** Molecular formulae of secondary metabolites not previously found in the Dictionary of natural products.

Strain	Molecular formula of Compounds unmatched in DPN	Comment
*Streptomyces* sp. A-28	C_16_H_18_O_9_	
	C_18_H_21_NO_6_	
*Streptomyces*sp. A-104	C_15_H_18_O_7_	
	C_15_H_20_O_5_	
*Streptomyces* sp. A-106	C_29_H_38_N_2_O_7_	
*Streptomyces* sp. A-123	C_12_H_16_ClNO_3_	
	C_47_H_64_O_13_	Related to pulvomycin
*Streptomyces*sp. A-124	C_8_H_7_N_5_O	
	C_23_H_18_N_4_O_6_	
	C_25_H_22_N_4_O_7_	
*Streptomyces* sp. A-125	C_16_H_19_NO_4_	Polyene UV
*Streptomyces* sp. A-145	C_43_H_54_N_2_O_12_	
	C_41_H_45_NO_12_	
	C_39_H_41_NO_10_	
*Streptomyces* sp. A-159	C_54_H_75_N_9_O_13_	New Ilamycin
*Streptomyces* sp. A-163	C_12_H_24_O_4_	
	C_18_H_16_O_4_	
	C_11_H_13_NO_2_	
	C_12_H_20_O_3_	
*Streptomyces*sp. A-177	C_15_H_9_Cl_2_NO_4_	New caboxamycin derivative^a^
	C_15_H_10_ClNO_4_	New caboxamycin derivative
*Streptomyces*sp. A-183	C_20_H_28_O_3_	
	C_22_H_31_NO_2_	
	C_14_H_26_O_5_	
*Micromonospora*sp. A-199	C_14_H_12_N_2_O_5_	Posible Diazepinomycin

^a^ Caboxamycin B (**1**) described in this article.

Concerning the sources of compounds, Table 5 displays the number of identified and unidentified compounds produced by each producing strain, and the results of meteorological analyses to know the sources and trajectories of the air masses causing the precipitation events in which they were isolated, estimated with a 5-day NOAA HYSPLIT Model, (Figure 2). According to backward transport trajectories analyses, the air masses mainly originated in the Arctic Ocean and travelled southward to downwind areas, such as the sampling places in Europe in a trajectory that also involves the Atlantic Ocean. As shown in Table 5, the unidentified molecules were produced by 12 strains, 11 *Streptomyces* and one *Micromonospora*, and were sampled in winter events mostly sourced around the North Pole. Nine unidentified molecules were obtained in events sourced in Svalbard archipelago, Norway (01/02/2015), four in the North Pole (31/01/2015), three in the White Sea (12/01/2016), two in Lofoten Islands, Norway (16/05/2013). The remaining events in which unidentified molecules were obtained were sourced in the Davies Strait, in the Arctic Ocean, three in the 21/01/2015 event, one in the 24/03/2015 and one in 14/02/2016. Also, 2 novel molecules were obtained in the 20/12/2015 event, in which the air mass at 3,000 m is sourced in the Davies Strait, whereas at 1,000 m originated in Mauritania and Canary Islands, and at 30 m were sourced in Spain (Iberian Peninsula).

**TABLE 5:** Sources of atmospheric Actinobacteria producing new natural products. Number of compounds, air masses sources and trajectories of the precipitation events. Air masses backward trajectories analyses were estimated as indicated in Figure 2.

	Number of compounds														
Strain	Identified	Undentified	Precipitation	Sampling date	Air masses trajectories (Figure 2)										
*Streptomyces* sp*.* A-4	5		Snow	25/02/2013	Arctic Ocean, White Sea, Sweden, Baltic Sea, Lituania, Denmark, England, Normandie (France), Cantabrian Sea										
*Streptomyces* sp. A-28	7	2	Rain	16/05/2013	Arctic Ocean, Lofoten Ilands (Norway), Iceland, Atlantic Ocean, North Spain (Galicia)										
*Streptomyces* sp. A-104	4	2	Rain	21/01/2015	Arctic Ocean (close to the magnetic pole) Baffin, Davies Strait, Greenland, Atlantic Ocean, Ireland, Cantabrian Sea										
*Streptomyces* sp. A-105			Rain	21/01/2015	Arctic Ocean (close to the magnetic pole) Baffin, Davies Strait, Greenland, Atlantic Ocean, Ireland, Cantabrian Sea										
*Streptomyces* sp. A-106	3	1	Rain	21/01/2015	Arctic Ocean (close to the magnetic pole) Baffin, Davies Strait, Greenland, Atlantic Ocean, Ireland, Cantabrian Sea										
*Streptomyces* sp. A-120	2		Hailstone	31/01/2015	Arctic Ocean, (North Pole), Atlantic Ocean, North Spain										
*Nocardiopsis* sp. A-121	6		Hailstone/Rain	01/02/2015	Arctic Ocean (close to North Pole), Svalbard (Norway), Iceland, Canada, Greenland, Atlantic Ocean, Cantabrian Sea										
*Streptomyces* sp. A-123	4	2	Hailstone/Rain	01/02/2015	Arctic Ocean (close to North Pole), Svalbard (Norway), Iceland, Canada, Greenland, Atlantic Ocean, Cantabrian Sea										
*Streptomyces* sp. A-124	4	3	Hailstone/Rain	01/02/2015	Arctic Ocean (close to North Pole), Svalbard (Norway), Iceland, Canada, Greenland, Atlantic Ocean, Cantabrian Sea										
*Streptomyces* sp. A-125	6	1	Hailstone/Rain	01/02/2015	Arctic Ocean (close to North Pole), Svalbard (Norway), Iceland, Canada, Greenland, Atlantic Ocean, Cantabrian Sea										
*Streptomyces* sp. A-145	6	3	Hailstone/Rain	01/02/2015	Arctic Ocean (close to North Pole), Svalbard (Norway), Iceland, Canada, Greenland, Atlantic Ocean, Cantabrian Sea										
*Streptomyces* sp. A-159	3	1	Rain	24/03/2015	Arctic Ocean, Canada, Greenland, Norway, Sweden, Denmark, Germany, Nederland, Belgium, France, Cantabrian Sea										
*Streptomyces* sp. A-163	6	4	Hailstone	31/01/2015	Arctic Ocean (North Pole), Atlantic Ocean, North Spain										
*Micromonospora* sp. A-176	7		Rain	20/12/2015	Arctic Ocean, Davies Strait, Atlantic Ocean, Mauritania, Portugal, Spain (including Canary Ilands)										
*Streptomyces* sp. A-177		2	Rain	20/12/2015	Arctic Ocean, Davies Strait, Atlantic Ocean, Mauritania, Portugal, Spain (including Canary Ilands)										
*Streptomyces* sp. A-182	7		Rain	12/01/2016	Arctic Ocean, White Sea, Russia, Finland, Sweden, Norway, Iceland, Atlantic Ocean, North Spain										
*Streptomyces* sp. A-183	14	3	Rain	12/01/2016	Arctic Ocean, White Sea, Russia, Finland, Sweden, Norway, Iceland, Atlantic Ocean, North Spain										
*Micromonospora* sp. A-199		1	Hailstone^a^	14/02/2016	Arctic Ocean, Davies Strait, Greenland, Iceland, Labrador (Canada), Atlantic Ocean										

^a^ Sarmiento-Vizcaíno et al., 2018.

### Structural elucidation of caboxamycin B

**FIGURE 5:**
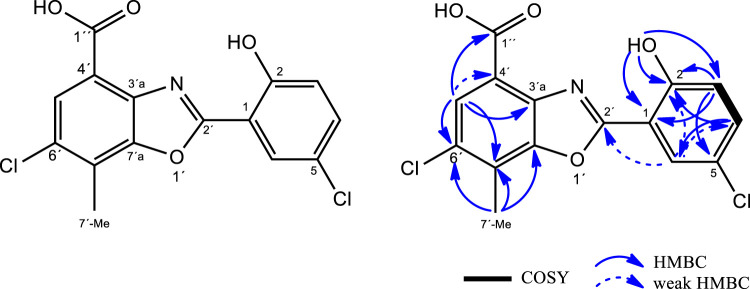
Structure of caboxamycin B (1) and key COSY/HMBC correlations.

LC-UV-HRMS analyses of extracts from *Streptomyces* sp. A-177 revealed the presence of two new natural products structurally related to caboxamycin, an antibiotic belonging to the benzoxazole family produced by a deep-sea *Streptomyces* isolated in the Canary Basin (Hohmann et al., 2009), and whose biosynthetic pathway was elucidated (Losada et al., 2017) (Table 4). One of these compounds, caboxamycin B (1), was isolated by HPLC as a peak with a characteristic UV spectrum (SI, Fig SX and its structure was determined as shown in Figure 5, based on HRMS and 1D/2D NMR spectroscopy. The (+)ESI-TOF spectrum of 1 (Supplementary Figure S1) showed a [M + H]^+^ ion at *m/z*337.9980, with an isotopic pattern corresponding to the presence of two chlorine atoms, indicative of the molecular formula C_15_H_9_Cl_2_NO_4_ (*Δ* = -0.30 ppm) which was not found in any natural products database. Interestingly, another ion present in the spectrum at *m/z*727.9016 and its isotopic distribution fitted very well with the molecular formula C_30_H_15_Cl_4_N_2_O_8_Fe ([2M-2H + Fe]^+^; calcd, 727.9005, *Δ* = +1.52 ppm), which suggests that **one** is able to complex iron, as previously reported for caboxamycin (Supplementary Figure S2).

The ^1^H-NMR spectrum of **1** (Supplementary Table S1, Supplementary Figure S3) showed four aromatic hydrogen signals, two doublets (δ_H_ 7.19 and 7.60 ppm; both with ^3^ *J*_H,H_ = 8.7 Hz) and two broad singlets (*δ*_H_7.96 and 8.13 ppm), as well as one singlet methyl at *δ*_H_2.66 ppm and one additional singlet at *δ*_H_11.66 ppm. Also, a weak signal as a broad singlet is observed at *δ*_H_13.51 ppm, suggesting the presence of a carboxylic acid group. Interpretation of the ^13^C-NMR (Supplementary Figure S4) and HSQC (Supplementary Figure S6) spectra confirmed the presence of four aromatic methines and one methyl group and further revealed the presence often quaternary carbons in the structure. These data, along with the molecular formula, clarified that proton signal at 11.66 ppm should be bound to heteroatom and further supported the presence of a carboxylate group in **1**.

The benzoxazole-type structure of one was fully elucidated based on COSY, HSQC and HMBC correlations (Supplementary Figures S5–S8). The COSY (Supplementary Figure S5) spectrum revealed the presence of a single spin system comprising vicinal hydrogens H-3 and H-4 (δ_H_ 7.19 and 7.60 ppm, respectively). HMBC correlations from H-3 to C-1 and C-5, from H-4 to C-2 and C-6, from H-6 to C-2, C-4 and C-2´ (all of them weak but genuine cross-peaks; SI, Fig SX)and from 2-OH to C-1, C-2 and C-3, together with the chemical shift of C-5 (*δ*_C_ 123.6 ppm), jointly established the presence of a 5-chloro-salicyclic acid moiety (Figure 5).

On the other hand, the 4-methyl-5-chloro-3-hydroxy-anthranilic acid moiety was determined based on HMBC correlations form H-5′ to C-3′a, C-7′ and C-1´´ as well as from 7′-Me to C-6′, C-7′ and C-7′a, along with the characteristic chemical shifts of C-3′a and C-7′a (δ_C_ 137.2 and 149.4 ppm respectively) (Hohmann et al., 2009), confirming the structure of caboxamycin B as shown in Figure 5.

## Discussion

We provide here further evidence of the potential of the atmosphere in the discovery of natural products of interest in medicine and biotechnology. Antibiotic producing members of three genera of the phylum *Actinobacteria* were herein isolated in Spain during precipitation events with prevalent Northern winds over a 3 years period. Taxonomic identification and phylogenetic analyses revealed that atmospheric isolates belong mainly to *Streptomyces* genus, the largest of the phylum, with 690 currently validated species, but also to the Actinobacteria genus *Micromonospora*, with 110 validated species, and genus *Nocardiopsis* with 46 validated species. Bearing in mind previous reports on precipitations from Western and NorthWestern winds (Sarmiento-Vizcaíno et al., 2018, 2021), the results provided here from events with Northern winds further increases the number of *Streptomyces* homologues producing bioactive natural products so far isolated from atmospheric precipitations to a total of 47, which represents a 6.8% of the of the total number of currently validated species in this genus (http://www.bacterio.net/streptomyces.html).

Since atmosphere is an extreme environment, very selective for microorganisms, isolation of Actinobacteria from precipitations resulted much easier than from traditional terrestrial sources. In this innovative approach sampling is a straightforward process, precipitations literally fall from the sky and can bring in a great diversity of novel strains from sources that are potentially out of reach. Although many samples were herein analyzed throughout the years to explore the diversity of Actinobacteria in atmospheric environments, for an efficient natural product discovery approach it would be more convenient to analyze a higher volume from a single precipitation event. This way of proceeding has been successfully reported when sampling a hailstone storm in the same geographical region, where 38 potential novel molecules were found (Sarmiento-Vizcaino et al., 2018).

Meteorological analyses revealed that the air masses involving 5 days backward trajectories point to an Arctic Ocean origin and their diverse downwind pathways. These Actinobacteria remain viable after their atmospheric transport by winds across oceans and continents and are dispersed *via* the atmosphere before they fall down by precipitation. These findings provide further support for the *Streptomyces* atmospheric dispersal model (Sarmiento-Vizcaíno et al., 2016, 2021 **),** which is herein extended to the genus *Micromonospora*. This cycle may have been occurring over geological time scales being of relevance for the biogeography and evolutionary history of these Actinobacteria.

In oceanic environments, among Actinobacteria associated to marine organisms, it has been reported that the dominant genera of producers of secondary metabolites of pharmaceutical relevance are *Streptomyces* (68%), followed by *Micromonospora* (6%) and *Nocardiopsis* (3%) (Chen et al., 2021). The strains herein reported are closely related to bioactive oceanic species isolated in the Cantabrian Sea, North Atlantic Ocean. Among *Streptomyces*, of particular interest are *S. cyaneofuscatus* homolog strains (A-123, A-125 and A-159) which are homologues to highly producers of biologically active compounds, isolated during an oceanographic expedition to the submarine Avilés Canyon, Cantabrian Sea (Sarmiento-Vizcaíno et al., 2017b), where novel natural products with antibiotic and cytotoxic activities were discovered (Ortiz-López et al., 2018; Rodríguez et al., 2018). In addition, *Micromonospora* and *Nocardiopsis* species, known as a source for structurally diverse novel products (Subramani and Sipkema. 2019), were also isolated herein. *Micromonospora* species were previously found in snow from Svalbard, Norway, in the Arctic Circle at 78° N (Amato et al., 2007) and also more than 30 isolates with *Micromonopora*-like phenotype were obtained in other precipitation events (Sarmiento-Vizcaíno, 2016). Furthermore, bioactive species of this genus were also found to colonize deep sea environments, such as the Avilés Canyon up to 4,700 depth, in cold waters of Arctic origin (Sarmiento-Vizcaíno, 2016; Sarmiento-Vizcaíno et al., 2017a), where a novel compound displaying cytotoxic activities against human tumour cell lines was discovered (Sarmiento-Vizcaíno et al., 2017b). Concerning *Nocardiopsis*, bioactive members of this genus were previously isolated in atmospheric precipitation events with prevalent Western winds (Sarmiento-Vizcaíno et al., 2021).

Comparative analyses of secondary metabolites detected herein with natural products databases, led to the identification of a total of 69 structurally diverse compounds. Concerning their biological activity, after a literature search, 52% of the identified natural products are antibiotics (both against Gram-positive and Gram-negative bacteria and against fungi) and 32% are antitumor/cytotoxic agents. There also compounds with anti-inflamatory, antiviral, antiparasitic, immunosuppresant, immunostimulator, neurprotective, hepatoprotective and other pharmaceutical properties (Table 3). Of great relevance are 25 compounds with molecular formulae not previously reported in Natural product Databases (Table 4), since they are candidates for drugs discovery. Taken into account previous data (Sarmiento-Vizcaíno et al., 2018, 2021), the results here provided increase up to 93 the number of unidentified compounds so far reported to be produced by atmospheric Actinobacteria.

As a proof of concept a novel caboxamycin, caboxamycin B, the second of a family, was herein obtained from cultures of *Streptomyces* sp. A-177, and its structure established after HPLC purification and HRMS and NMR analysis. This new molecule is structurally related to caboxamycin, an antibiotic of the benzoxazole family initially obtained in the Canary Basin (Hohmann et al., 2009) and later on from *Streptomyces halstedii* M-204, asociated to an equinoderm in the Avilés Canyon (Sarmiento-Vizcaíno et al., 2017a). Remarkably, caboxamycin B constitutes the first halogenated caboxamycin reported in the literature. Our findings provide further support of the potential of atmospheric-derived Actinobacteria in the discovery of pharmaceutical natural products, particularly antibiotics, which are urgently needed for fighting against pathogenic bacteria resistant to antibiotics in currently in clinical use.

## Conclusion

Identification of Actinobacteria in precipitation events with prevalent Northern winds in Spain reveals that, besides *Streptomyces* and *Nocardiopsis* species previously reported in samples from Western and North Western winds, bioactive members of the genus *Micromonospora* are also present in atmospheric environments. In total, almost a hundred of potential novel natural products have been establish in these Actinobacteria isolated from precipitation events with different winds so far. These findings confirm that atmospheric Actinobacteria deserve further research, since they produce a remarkable diversity of bioactive compounds of pharmacological and biotechnological relevance. In this line of thought, the discovery of a novel molecule structurally related to the antibiotic caboxamycin, such as caboxamycin B, is to the best of our knowledge the first novel natural product reported in a *Streptomyces* strain isolated from the atmosphere.

## Data Availability

The datasets presented in this study can be found in online repositories. The names of the repository/repositories and accession number(s) can be found in the article/Supplementary Material.
