# Supplementary material for: Natural products, including a new caboxamycin, from Streptomyces and other Actinobacteria isolated in Spain from storm clouds transported by Northern winds of Arctic origin
Source: Front Chem. 2022 Nov 3;10:948795. doi: 10.3389/fchem.2022.948795 (PMC9669575; doi:10.3389/fchem.2022.948795)
Supplement: Supplementary file 1 [file DataSheet2.DOCX]

**Supplementary Material 2**

**List of contents**

**Figure S1.** a) HPLC-DAD chromatogram of caboxamycin B (**1**) (UV 210 nm). b) UV spectrum of **1**. c) HRESIMS(+)-TOF spectra (overview) of **1**

**Figure S2.** [M+H]^+^ and [2M-2H+Fe]^+^ ions in the HRESIMS(+)-TOF spectrum of **1** (zoomed regions)

**Figure S3.** ^1^H NMR spectrum of caboxamycin B (**1**)

**Figure S4.** ^13^C NMR spectrum of caboxamycin B (**1**)

**Figure S5.** COSY spectrum of caboxamycin B (**1**)

**Figure S6.** HSQC spectrum of caboxamycin B (**1**)

**Figure S7.** HMBC spectrum of caboxamycin B (**1**)

**Figure S8.** Expansion of the HMBC region corresponding to H-6 of caboxamycin B (**1**)

**Table S1.** NMR Data (500 MHz, DMSO-*d_6_* at 24 C) of caboxamycin B (**1**)

a)

b)

c)

**Figure S1.** a) HPLC-DAD chromatogram of caboxamycin B (**1**) (UV 210 nm). b) UV spectrum of **1**. c) HRESIMS(+)-TOF spectra (overview) of **1**

**Figure S2.** [M+H]^+^ and [2M-2H+Fe]^+^ ions in the HRESIMS(+)-TOF spectrum of **1** (zoomed regions)

**Figure S3.** ^1^H NMR spectrum of caboxamycin B (**1**)

**Figure S4.** ^13^C NMR spectrum of caboxamycin B (**1**)

**Figure S5.** COSY spectrum of caboxamycin B (**1**)

**Figure S6.** HSQC spectrum of caboxamycin B (**1**)

**Figure S7.** HMBC spectrum of caboxamycin B (**1**)

**Figure S8.** Expansion of the HMBC region corresponding to H-6 of caboxamycin B (**1**)

**Table S1.** NMR Data (500 MHz, DMSO-*d_6_* at 24 C) of caboxamycin B (**1**)

| position | *δ*_C_, mult | *δ*_H_, mult (*J* in Hz) |
| --- | --- | --- |
| 1 | 111.3, C |  |
| 2 | 157.0, C |  |
| 2-O*H* |  | 11.66, s |
| 3 | 119.4, CH | 7.19, d (8.7) |
| 4 | 134.2, CH | 7.60, d (8.7) |
| 5 | 123.6, C |  |
| 6 | 126.9, CH | 8.13, br s |
| 2´ | 162.8, C |  |
| 3´a | 137.2, C |  |
| 4´ | 120.0, C |  |
| 5´ | 127.4, CH | 7.96, br s |
| 6´ | 130.0, C |  |
| 7´ | 124.9, C |  |
| 7´-Me | 13.4, CH_3_ | 2.66, s |
| 7´a | 149.4, C |  |
| 1´´ | 164.5, C |  |
| -COO*H* |  | 13.51, br s |
