# Supplementary material for: Natural products, including a new caboxamycin, from Streptomyces and other Actinobacteria isolated in Spain from storm clouds transported by Northern winds of Arctic origin
Source: Front Chem. 2022 Nov 3;10:948795. doi: 10.3389/fchem.2022.948795 (PMC9669575; doi:10.3389/fchem.2022.948795)
Supplement: Supplementary file 2 [file DataSheet1.DOCX]

**Supplementary Material 2**

*Streptomyces* sp. A-4

*Streptomyces* sp. A-28

*Streptomyces* sp. A-104

*Streptomyces* sp. A-105

*Streptomyces* sp. A-106

*Streptomyces* sp. A-120

*Nocardiopsis* sp. A-121

*Streptomyces* sp. A-123

*Streptomyces* sp. A-124

*Streptomyces* sp. A-125

*Streptomyces* sp. A-145

*Streptomyces* sp. A-159

*Streptomyces* sp. A-163

*Micromonospora* sp. A-176

*Streptomyces* sp. A-177

*Streptomyces* sp. A-182

*Streptomyces* sp. A-183

*Micromonospora* sp. A-199
